# Neuromorphic Model of Reflex for Realtime Human-Like Compliant Control of Prosthetic Hand

**DOI:** 10.1007/s10439-020-02596-9

**Published:** 2020-08-20

**Authors:** Chuanxin M. Niu, Qi Luo, Chih-hong Chou, Jiayue Liu, Manzhao Hao, Ning Lan

**Affiliations:** 1 Laboratory of Neurorehabilitation Engineering, School of Biomedical Engineering, Shanghai Jiao Tong University, 1954 Hua Shan Road, Med-X Research Institute, Rm 405 (South), Shanghai, China; 2 Department of Rehabilitation Medicine, Ruijin Hospital, School of Medicine, Shanghai Jiao Tong University, Shanghai, China; 3 Institute of Medical Robotics, Shanghai Jiao Tong University, Shanghai, China

**Keywords:** Biomimetic control, Prosthetic hand, Neuromuscular reflex, Neuromorphic modeling, Electromyography (EMG)

## Abstract

Current control of prosthetic hands is ineffective when grasping deformable, irregular, or heavy objects. In humans, grasping is achieved under spinal reflexive control of the musculotendon skeletal structure, which produces a hand stiffness commensurate with the task. We hypothesize that mimicking reflex on a prosthetic hand may improve grasping performance and safety when interacting with human. Here, we present a design of compliant controller for prosthetic hand with a neuromorphic model of human reflex. The model includes 6 motoneuron pools containing 768 spiking neurons, 1 muscle spindle with 128 spiking afferents, and 1 modified Hill-type muscle. Models are implemented using neuromorphic hardware with 1 kHz real-time computing. Experimental tests showed that the prosthetic hand could sustain a 40 N load compared to 95 N for an adult. Stiffness range was adjustable from 60 to 640 N/m, about 46.6% of that of human hand. The grasping velocity could be ramped up to 14.4 cm/s, or 24% of the human peak velocity. The complaint control could switch between free movement and contact force when pressing a deformable beam. The amputee can achieve a 47% information throughput of healthy humans. Overall, the reflex-enabled prosthetic hand demonstrated the attributes of human compliant grasping with the neuromorphic model of spinal neuromuscular reflex.

**Electronic supplementary material:**

The online version of this article (10.1007/s10439-020-02596-9) contains supplementary material, which is available to authorized users.

## Introduction

Upper limb amputation would lead to permanent loss of hand function in amputees. The needs for recovery, compensation and replacement of their hand functionality have motivated generations of neural prosthetic technologies.^44^,^59^,^65^,^82^ Despite the advancement of neural prosthetic hands in the past decades, rejection of prosthetic hands remains high.^6^,^66^ A primary challenge for existing prosthetic hands is that they lack the compliant properties in the grasp control, such that the grasping behavior is indifferent to the stiffness or brittleness of the object. As a result, the amputee may find it difficult to attend to the mechanical subtlety of grasped objects.^13^,^29^,^59^ Therefore, an inevitable question of transcending prosthetic hand is how human-like compliant control can be reified on the device. 

When interacting with real-world objects, a prosthetic hand is expected to adapt its behavior commensurate with the object, especially for objects that are deformable, crispy, or irregular in shape. In able-bodied individuals, nuances of an object are perceived through an interplay among visual, tactile, and proprioceptive information^32^; In amputees, however, both tactile and proprioceptive feedback are compromised at their stumps,^12^,^19^,^91^ leaving visual feedback an indirect source for the calculation of grasping force.^71^ In this scenario, if signals about hand gesture can be engineered into biomimetic forms such as spike trains, it may be possible to emulate the flow of proprioceptive information,^61^ which can be integrated into an artificial controller mimicking human reflex. This is a fundamentally different approach for prosthetic control compared to most existing applications, which typically model the controllers as linear-time-invariant systems.^43^,^46^,^57^

The role of reflex in human movement has been demonstrated in a long history of scientific literature.^31^,^52^,^63^,^68^,^76^ Computational models remain central to the reconstruction of reflex for prosthetic hand. Mammalian experiments have revealed principles instrumental for neural control of movements: motor units with patterned recruitment order,^37^ spinal-level neural circuitry,^1^,^67^ dynamics of muscle spindle,^54^ muscle with viscoelastic properties^90^ and so on. In case of stretch reflex, the stretching is sensed by the muscle spindle, transduced into excitatory spikes in the afferents, and it eventually increases the muscle force to resist the stretch. This reinforcement is added on to the inherent stiffness of muscles to further adjust force control.^60^ Reverse engineering of muscle spindle started from the pioneering work on animal neurography,^54^ followed by computational modeling of spindle,^56^ the functional implication,^48^ the addition of spiking interface and real-time emulation,^62^ and recently a “model-in-the-loop” application that demonstrated its sufficiency in limb control.^61^

One of the challenges with hand prosthesis is how to faithfully execute movement intention of the amputee. EMG signals have been generally adopted to control the prosthetic hand in a wide range of schemes, including proportional control,^3^,^33^,^34^,^72^,^79^ regression control,^40^,^58^,^81^ on-off control,^80^ finite state machine control,^15^,^46^ pattern recognition-based control,^4^,^22^,^77^,^86^,^88^ postural control,^73^,^74^,^89^ etc. An alternative approach for myocontrol is to decode the latent control variable behind raw EMG using biology-inspired models. In most cases, the EMG signal was modeled as amplitude-modulated band-limited noise,^16^,^18^ and therefore the EMG signal was filtered and decoded for myocontrol by algorithms that extract the amplitude envelope.^39^ Sanger demonstrated a new Bayesian model for EMG that described the motor intent as a stochastic process with jumping^70^ for myocontrol applications.^7^

This study proposes a new methodology of real-time control for cable-driven prosthetic hands using a human-like reflex model. In specific, we seek to reconstruct biomimetic reflex on a prosthetic hand, which presumably enables a natural translation between motor command and hand movement. The biomimetic reflex includes spiking neurons, skeletal muscles, muscle spindles, spinal circuitries, etc. All components in the reflex have been derived from well-established previous experiments and modeling work. The real-time closed-loop execution of reflex is enabled by neuromorphic hardware, a customized architecture of VLSI (very-large-scale-integrated-circuit) that parallelizes large-scale model computation. The goal of this study is to verify the feasibility of model-based control for hand prosthesis. We hypothesize that with the help of biomimetic reflex, a cable-driven prosthetic hand could exhibit rich behaviors of human reflexes in response to constraints from the environment and task. Results will justify whether the model-based control approach may have significant benefits for the motor performance of prosthetic hand.

## Materials and Methods

In this study, we proposed a model-based control strategy for prosthetic hand, which was inspired by the neuromuscular control of upper-extremities in human. The model emulated a monosynaptic reflex loop by implementing elements known to be important in biological neural control. Surface EMG from wrist muscles was used to activate a torque motor, which pulled a cable to create hand movements. Wrist flexors were chosen because those muscles were still functioning after fore-arm amputation, and wrist flexors were easily recorded using sEMG for myocontrol.^25^,^27^,^30^,^36^ Using this methodology, we were able to build a prosthetic hand platform that enabled us to ask whether introducing principles from human sensorimotor control may benefit the performance of hand prosthesis.

The following parts are presented in the following order: first the build-up of the cable-driven prosthetic as the mechanical plant; second the detailed descriptions of the model-based controller implementing biomimetic reflex, especially how components were modeled following the flow of signals; third how the model was emulated on neuromorphic hardware.

### Cable-Driven Prosthetic Hand Operated by Wrist-Flexor EMG

Prosthetic hands with cable-driven designs are favored for model-based control. This is because cable-driven mechanisms are compatible with tendon-driven fingers in back-drivability; also the translational movement on a cable is compatible with the lengthening of a muscle.^85^ Without loss of generality, we demonstrate model-based control using an open-source design of cable-driven hand (InMoov).^41^ The hand was 3D-printed and assembled for under 100 U.S. dollars. Other candidate designs of cable-driven hands include Ada hand, Brunel, Dextrus, etc.^9^,^11^,^14^,^64^,^78^; pneumatically-driven hands are also acceptable.^20^,^83^ Hands with gear or linkage transmission,^17^,^45^,^50^,^53^,^84^ however, are not directly applicable for model-based control, because simulating pull-only muscle tension can be non-trivial.

**Figure 1:**
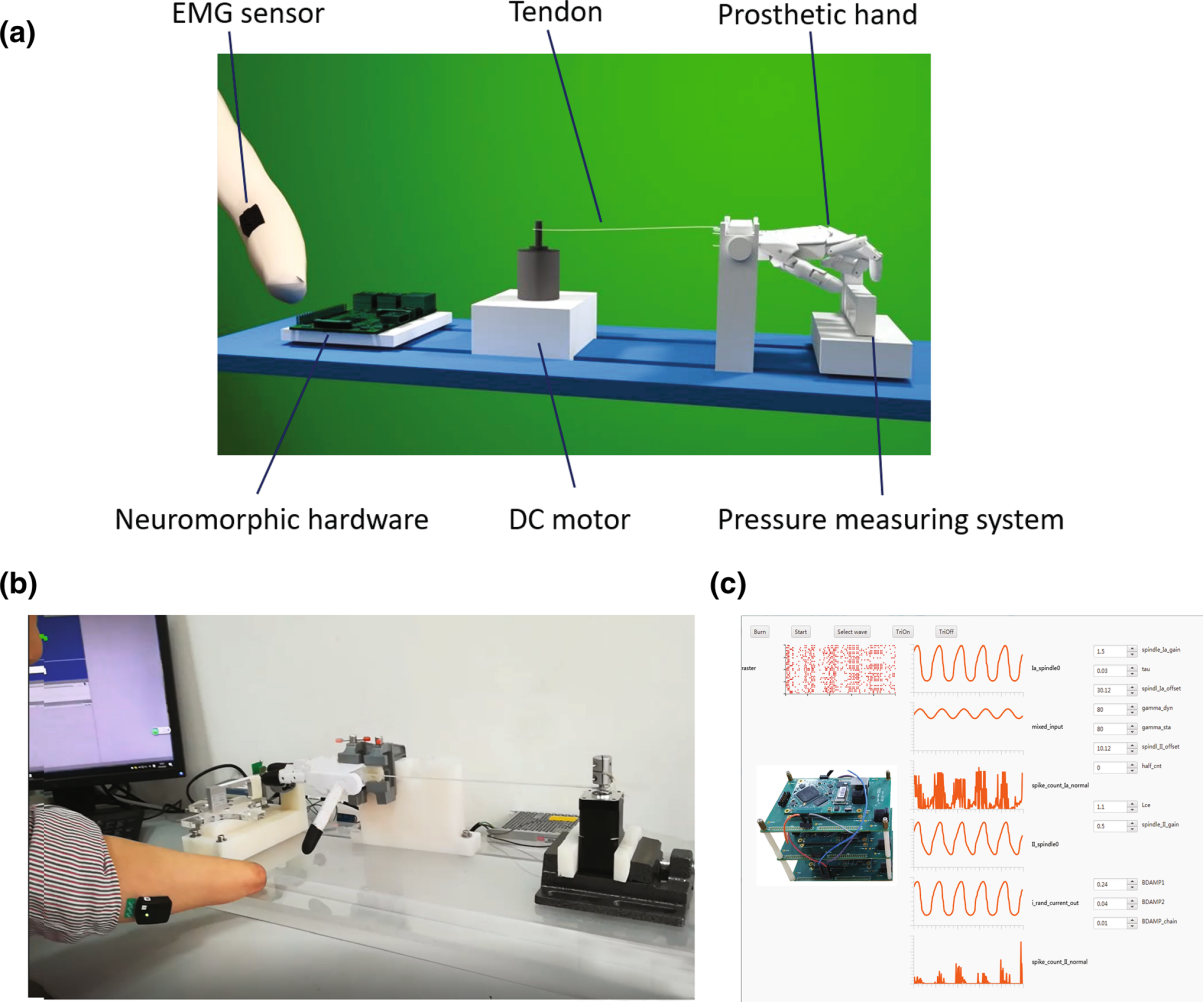
(a) The design of a tendon-driven prosthetic hand actuated by a torque motor, which executes commands issued from the stump EMG modulated by neuromorphic hardware. One piece of neuromorphic hardware is illustrated. A force transducer measures the downward pressure from the index finger. (b) Scene of a forearm amputee operating the cable-driven prosthetic hand with his flexor carpi ulnaris EMG. (c) The interfacing software configures the parameters of the reflex model and displays the internal statuses of emulated reflex. A total of 3 stacked neuromorphic boards are shown, which have formed a biomimetic reflex loop.

In our implementation, hand joints were pulled using high density Polyethylene (PE) cables as proxies of human tendon, each of which simultaneously flexed the metacarpophalangeal (MCP), Distal Interphalangeal (DIP), and proximal interphalangeal (PIP) joints. The cables were attached to the shaft of a DC torque motor (PD2-C42, Nanotec Electronics GmbH & Co.KG, Germany) communicated through a CANopen interfacing card (ECAN-IT, Guangcheng Technology Co., Ltd., Shenyang, China). The motor generated a torque resulting in a tension on the cable. The main control loop of the prosthetic hand was coordinated on a PC (Intel Core i7-8700CPU, 3.20 GHz, 16 GB Memory, Microsoft Windows 10 64-bit) at 100Hz sampling rate. Only finger flexion was activated by the user; finger extension was passively applied by an extending spring in each joint. Figure 1 shows an illustration and the work scene of the cable-driven prosthetic hand.

### Model-Based Controller with Biomimetic Reflex

**Figure 2:**
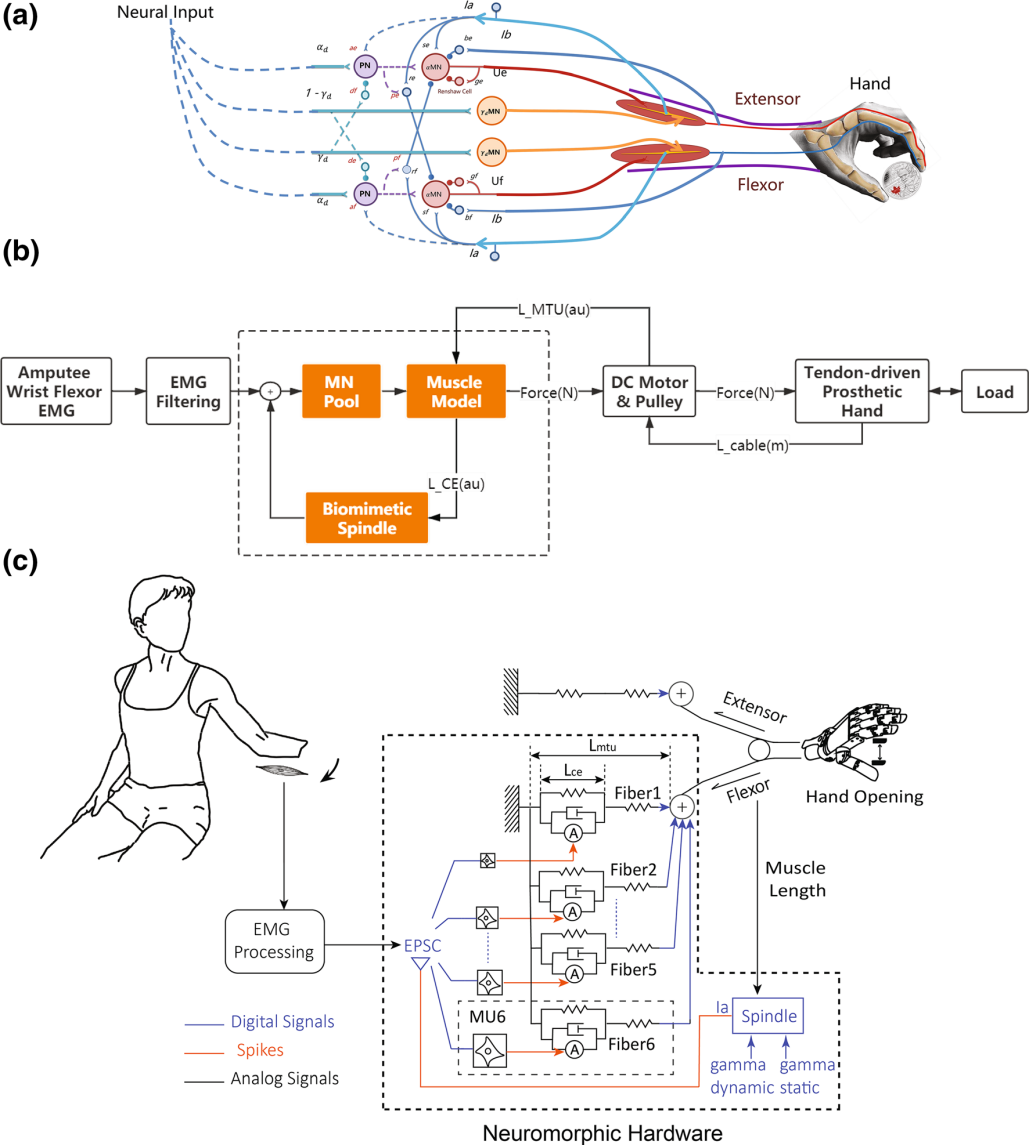
(a) Elements of a monosynaptic spinal reflex that are relevant to the neural control of hand movement. A finger joint is stretched opposingly by a flexor and an extensor, each of which is activated by a pool of motoneurons under the continuous modulation of spindle feedback. (b) Block diagram of the model-based biomimetic controller. Wrist EMG is filtered using a nonlinear Bayesian algorithm, which eventually establishes the model-produced force on the DC motor; proprioceptive information is deduced from the DC motor to populate muscle spindle. (c) Detailed architecture of the model-based controller. An excitatory post-synaptic current (EPSC) drives 6 motoneuron pools of different sizes, which produces a superimposed force output to pull the cable. Dashed region is implemented on the neuromorphic hardware.

The neuromuscular components of human hand control at the level of spinal-cord are illustrated in Fig. 2a. We focus on the spinal level of human nervous system because the supra-spinal structures are not lesioned through amputation. In a typical joint (e.g. the metacarpophalangeal joint) rotated by an antagonistic pair of muscles, muscle contraction produces the force transferred via tendons attached to the joint. Muscle contraction is determined first by the spiking excitations from motoneurons (alpha and gamma), and the contraction is also modulated by proprioceptive feedback from muscle spindles.

The overall architecture of the controller is shown in Fig. 2b. Colored blocks represent the key models from neurophysiology of reflex, meaning that their dynamic behavior should reflect a mammalian reflex loop, which includes a pool of motoneurons, a skeletal muscle, and associated muscle spindles. Raw EMG signals are filtered into an alpha motor command activating the motoneuron pool, which is then converted into a force of contraction through muscle model, followed by a DC motor to establish the calculated force; meanwhile, the calculation of muscle force is constantly adjusted by a biomimetic spindle.

### Definitions and Responsibilities of Models in Biomimetic Reflex

Details about a biomimetic reflex are shown in Fig. 2c. The alpha motor command is filtered from sEMG on the wrist flexor (normally flexor carpi ulnaris, FCU) from the amputee. The motor command enters the biomimetic reflex loop as an excitatory post-synaptic current (EPSC), which is distributed to several pools of spiking motoneurons. Spiking outputs from the motoneurons are subsequently converted to excitatory drive to Hill-type models of skeletal muscle. The muscle model calculates a muscle force that will eventually be established by the DC motor. Information about muscle lengthening is sent back to a model of muscle spindle, which subsequently produces excitatory afferents that loop back to the motoneuron pool.

The mathematical definitions of models are listed in Table 1. It is required that adjacent models must communicate with compatible types of signal. And all interfacing signals should be measurable in reality, such that the validity of model-based control can be verified against literature.

**Table 1:** Mathematical formulation of components in the model-based controller.

Component	Formulation	Note
Alpha motor command	$\alpha = f_{\text{alpha}} \left( {\text{EMG}} \right)$	$\alpha \in \left[ {0,1} \right]$ is the normalized alpha motor command
Motoneuron model	$S_{\text{mn}}^{*} = f_{\text{Izhikevich}} \left( {k\alpha } \right)$	$k$ is a scaling factor that converts unit-less alpha motor command to transmembrane current (mA) required by Izhikevich neuron model
Muscle model	$T = f_{\text{Hill}} \left( {S^{*} ,L,\dot{L}} \right)$	
Spindle model	$S_{\text{af}}^{*} = f_{\text{spindle}} \left( {L,\dot{L},\varGamma_{\text{dyn}} ,\varGamma_{\text{sta}} } \right)$	
Synapse model	$I = f_{\text{synapse}} \left( {S^{*} } \right)$	See Appendix (Ref. 28)

#### Model for Alpha Motor Command

Alpha motor command is the entrance to the model-based controller (Fig. 2b). We propose to decode the alpha motor command by filtering the wrist flexor EMG from the amputee. Wrist flexors are superficial in anatomy so they are widely used in myocontrol applications.^25^,^27^,^30^,^36^ The filtering was done using a nonlinear Bayesian algorithm that has demonstrated its advantage in myocontrol applications.^7^

#### Model of Spiking Neuron and Motoneuron Pool

Spiking neurons are implemented using the Izhikevich approximation^42^ to the classic Hodgkin–Huxley model.^38^ In the biomimetic reflex, we include 768 alpha-motoneurons divided into 6 pools representing motor units of 6 various sizes (Fig. 2c). The recruitment order in motoneuron pools naturally emerge due to Henneman’s size-principle.^37^ Since each motoneuron pool contains 128 neurons, their EPSCs are superimposed with a Gaussian noise to allow 128 neurons fire at similar rate but with different timing. The number of motoneuron was determined by balancing the innervation number of a typical mammalian muscle^8^ and the maximum number allowed by the hardware.^61^ More details about neuron modeling are listed in Appendix.

#### Model of Skeletal Muscle with Access to Proprioception

**Figure 3:**
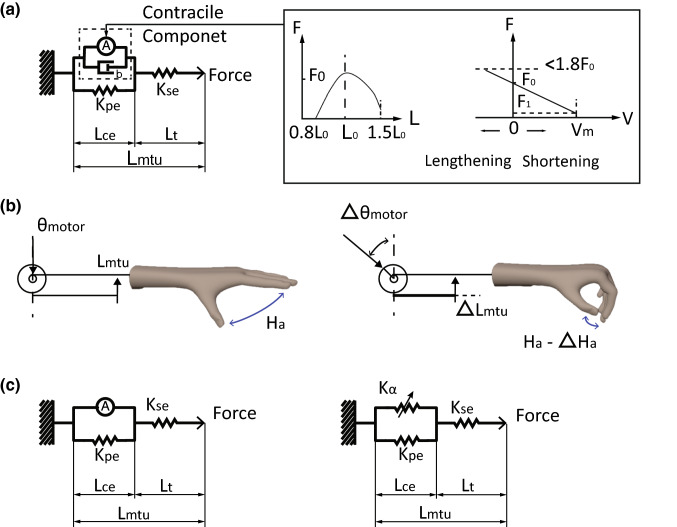
(a) Modified Hill-type muscle model with force-length and force-velocity characteristics. The active component was modified to allow for spiking inputs. (b) Conversion of finger flexion to muscle length. An alpha motor command rotates the electric motor $(\Delta \theta_{\text{motor}} )$, which produces a translational movement on the cable ($\Delta L_{\text{mtu}}$), which reduces the opening aperture of the hand ($\Delta H_{a}$). (c) Conversion from $L_{\text{mtu}}$ to $L_{\text{ce}}$ for muscle spindle. By ignoring the damping in the contractile component, the Hill-type model reduces to the left scenario. We resolve $L_{\text{ce}}$ by approximating the active component as a stiffness-adjustable spring ($K_{\alpha }$, right).

Skeletal muscles are implemented using a Hill-type model (Fig. 3a). The formulation is based on a version with active twitch by Shadmehr and Wise,^75^ and a modification that can receive spike train as the input.^61^ Therefore, the muscle model converts α-motoneuron spikes into muscle force, depending on the muscle’s history of length and lengthening velocity. All 6 motoneuron pools are connected to the same type of muscle fiber that was capable of twitching at 30 ms time constant. It is possible, given more on-chip resources, to differentiate muscle fibers by diversifying the time constants of twitch.^26^

The force-length and force-velocity properties are introduced by multiplying a length-sensitive term ($F_{L}$) and a velocity-sensitive term ($F_{V}$) to the active muscle force ($F_{A}$), as is described by Zajac.^90^ In specific, the force-length term is given by:

$$ F_{L} = \left\{ {\begin{array}{ll} 0 \\ { - 4.095L^{2} + 8.190L - 3.071} \\ { - 1.67L^{2} + 2.672L } \\ \end{array} } \right.\begin{array}{ll} {\left( {L\left\langle {0.5 {\text{or}} L} \right\rangle 1.6} \right)} \\ {\left( {0.5 \le L < 1} \right)} \\ {\left( { 1 < L \le 1.6} \right)} \\ \end{array} $$

The force-velocity term is given by:

$$ F_{V} = \frac{{F_{1} - F_{0} }}{{V_{m} }}*V + F_{0} ,     (F_{V} < 1.8F_{0} ) $$

Other details about the Hill-type model of muscle are provided in the Appendix.

#### Model of Proprioceptive Information Based on Motor Rotation

Muscle length and lengthening velocity are critical information in the biomimetic reflex: they are the source of proprioception that should be explicitly provided to muscle spindle, and they must be accessible by the muscle itself to engage force-length and force-velocity properties. Both the length of musculotendinous unit ($L_{\text{mtu}}$) and length of contractile element ($L_{\text{ce}}$) and are captured in the biomimetic reflex.

$L_{\text{mtu}}$ is equivalent to the translational displacement of the cable, which is measured by a rotational transducer on the DC motor (Fig. 3b):

1 $$ \begin{aligned} L_{\text{mtu}} \left( t \right) & = L_{\text{mtu}} \left( 0 \right) - \Delta L_{\text{mtu}} \\ & = L_{\text{mtu}} \left( 0 \right) - r\Delta \theta_{\text{motor}} \\ \end{aligned} $$

meaning that the instantaneous $L_{\text{mtu}} \left( t \right)$ equals the length of the initial cable minus the cable length coiled on the winder. $L_{\text{mtu}} \left( 0 \right) = 39.7\,{\text{cm}}$ denotes the initial length of the cable, as determined by the placement of the motor on anatomical socket; $L_{\text{mtu}} \left( 0 \right)$ is also similar to the length of a typical FCU MTU (38.3 cm).^24^$r = 0.25\,{\text{cm}}$ denotes the radius of the cable winder, which is chosen to allow for adequate travel on the cable (~ 1.57 cm for 360° of rotation) and maintain the ability of weight bearing (30 N at the fingertip).

$L_{\text{ce}}$ is the key input to the muscle spindle model as given by:

2 $$ L_{\text{ce}} \left( t \right) = L_{\text{ce}} \left( 0 \right) - \Delta L_{\text{ce}} $$

When the finger is moving freely without contact of object, $\Delta L_{\text{ce}}$ and $\Delta L_{\text{mtu}}$ are both equivalent to the translational displacement on the cable, and therefore it is straightforward to acquire $\Delta L_{\text{ce}} \left( t \right)$ from motor rotation. During contact, nevertheless, $\Delta L_{\text{ce}}$ is simultaneously affected by the active muscle force A, passive damping b, the parallel elastic component $K_{\text{pe}}$, and most importantly the deformation of the object. It is no longer trivial to acquire $\Delta L_{\text{ce}}$. Our approximation of $L_{\text{ce}}$ is described in the next section.

#### Approximation of Length of Contractile Element

When the fingers are in contact with an object, we ignore the effect of muscle damping due to relative slow lengthening of the muscle (Fig. 3b). According to the force-length curve,^55^ the active component in Hill-type muscle model is equivalent of a spring with adjustable stiffness $K_{\alpha }$ (Fig. 3c). Therefore, the length of contractile element is given by:

3 $$ L_{\text{ce}} = \frac{{K_{\text{se}} }}{{K_{\text{se}} + K_{\text{pe}} + K_{\alpha } }} *L_{\text{mtu}} $$

The adjustable stiffness $K_{\alpha }$ can be inferred from the force-length relationship as follows. We restrict the range to $L_{\text{mtu}} \le L_{\text{mtu}}^{0}$, such that only positive $K_{\alpha }$was effective.

4 $$ K_{\alpha } = \left\{ {\begin{array}{*{20}c} {\left( { - 57.76\alpha + 60.24} \right)*L_{\text{mtu}} + 57.41\alpha - 12.28,    L_{\text{mtu}} \le L_{\text{mtu}}^{0}  } \\ {\left( { - 14.61\alpha + 15.23} \right)*L_{\text{mtu}} + 14.47\alpha - 32.31,    L_{\text{mtu}} > L_{\text{mtu}}^{0} } \\ \end{array} } \right. $$

Therefore, the real length of $L_{\text{ce}}$ can be obtained as:

5 $$ L_{\text{ce}} = \left\{ {\begin{array}{*{20}c} {\frac{{L_{\text{mtu}} *K_{\text{se}} }}{{K_{\text{se}} + K_{\text{pe}} + \left( { - 57.761\alpha + 60.241} \right) *L_{\text{mtu}} + 57.405\alpha - 12.276}} ,L_{\text{mtu}} \le L_{\text{mtu}}^{0}  } \\ {\frac{{L_{\text{mtu}} *K_{\text{se}} }}{{K_{\text{se}} + K_{\text{pe}} + \left( { - 14.611\alpha + 15.238} \right) *L_{\text{mtu}} + 14.465\alpha - 32.307}}, L_{\text{mtu}} > L_{\text{mtu}}^{0} } \\ \end{array} } \right. $$

#### Model of Muscle Spindle and Proprioceptive Feedback

Proprioceptive feedback was provided by implementing the well-known model^56^ for muscle spindle. The $L_{\text{ce}}$ provided to the spindle model is normalized to the resting length $L_{\text{MTU}}^{0} = 23.6\,\,{\text{cm}}$ for FCU.^24^ The model characterized how nuclear bag and nuclear chain fibers produce Group Ia and Group II afferents; and influences from gamma motoneurons were also modeled. In the closed-loop control, only Group Ia afferents were included in the feedback loop, because the connections from Group II are not as clearly defined in monosynaptic circuitry. See Appendix for details on the spindle model.

### Real-Time Emulation of Biomimetic Reflex on Neuromorphic Hardware

The time-consuming components in the model-based controller are programmed on neuromorphic hardware, constructed under a customized VLSI architecture (implemented on Xilinx Spartan-6 FPGA). The neuromorphic design has been shown its advantage that allows large volume of spiking neurons to be evaluated in parallel without sacrificing speed.^61^ In addition, numerical floating-point calculations required by models are difficult to run at > 1 kHz on CPU. At least two Spartan-6 chips are required to enable an afferented muscle, one for motoneuron and muscle, the other for muscle spindles and their spiking afferents. The chips are connected by wires transmitting in raw spikes (Fig. 1c). These calculations are accelerated using combinatorial logics on FPGA. As a result, the biomimetic reflex managed to install a total of 768 motoneurons, 1 muscle spindle with 128 spiking afferents, and a Hill-type muscle model, all running with > 1 kHz refresh rate (Fig. 1c).

### Experiments for Verification of Hand Functionality

Three categories of experiments were conducted to validate the functionality of reflex-enabled control of prosthetic hand:

1. Basic functionality without human operation
2. Human-operated performance without object interaction
3. Human-operated performance with object interaction

Hand aperture was monitored using 240 fps slow-motion videos with a ruler in the scene. Details about the experiments are explained below in the Results. Signals were processed using MATLAB R2016b (MathWorks, Natick, MA). All statistical analyses were conducted using R (version 3.5.2).

## Results

### Experiment 1: Basic Functionality Without Human Operation

#### Hand Aperture is Linearly Controlled by Alpha Motor Command

**Figure 4:**
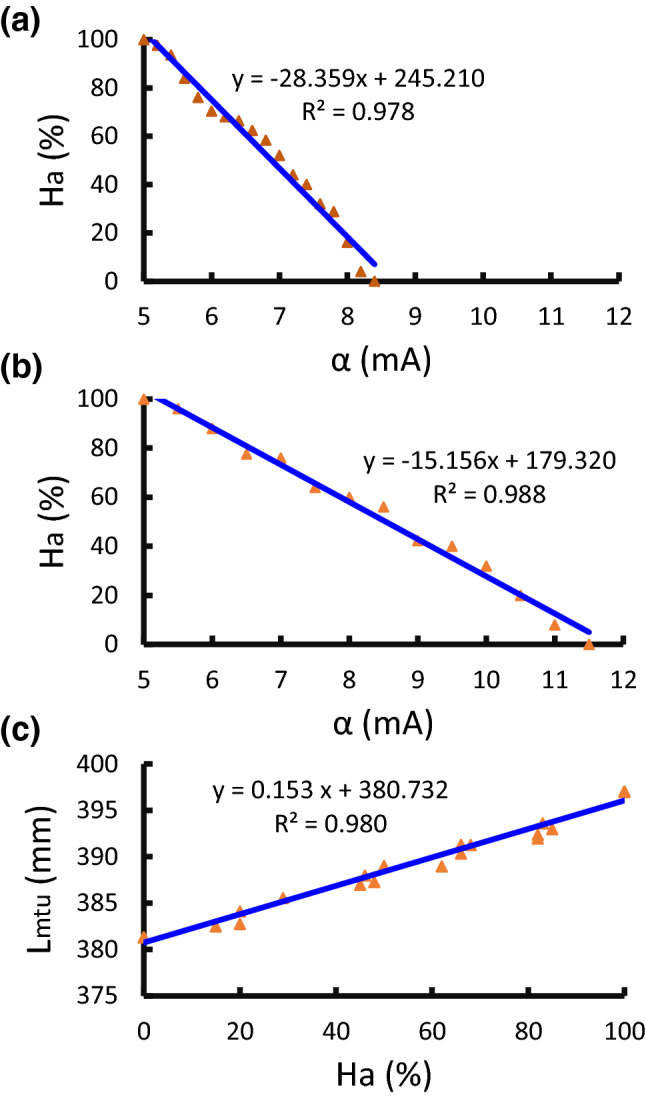
Experiment 1, hand closing in response to alpha motor command. (a) Three fingers (thumb, index, middle) were driven, the hand fully closed when the alpha motor command reached 8.4 mA. The linear relationship between hand opening aperture ($H_{a}$) and alpha command ($\alpha$) was significant. (b) All five fingers were driven, the hand fully closed when the alpha motor command reached 11.5 mA. The linear relationship between hand $H_{a}$ and $\alpha$ was also significant. (c) Linear relationship between muscle length ($L_{\text{mtu}}$) and aperture ($H_{a}$) as expected from the cable-driven design.

We first tested the ability of the biomimetic prosthetic hand for maintenance of an opening aperture ($H_{a}$). The hand was fully extended ($H_{a} = 100\%$) at the beginning. Then a certain level of alpha command was sent to the biomimetic model, which closed the hand to a steady-state aperture. The relationship between $H_{a}$ and alpha is shown in Fig. 4. Each data point was measured when the hand had stabilized at an aperture, then the hand was released to full extension for the next level of alpha command. When three fingers (thumb, index, middle) were pulled by the torque motor (Fig. 4a), the hand fully closed when the alpha motor command reached 8.4 mA. The linear relationship between $H_{a}$ and alpha was significant ($p < 0.05$, $R^{2} = 0.978$). When all five fingers were pulled (Fig. 4b), it required 11.5 mA alpha motor command to fully close the hand following a linear correlation ($p < 0.05$, $R^{2} = 0.988$). In both situations the hand was not moving with an alpha command less than 5 mA, most likely due to the initial torque required for the motor to move. Figure 4c shows that the opening aperture $H_{a}$ has a linear relationship with $L_{\text{mtu}}$ ($p < 0.05$, $R^{2} = 0.980$).

### Experiment 2: Human-Operated Performance Without Object Interaction

#### Hand Closing at Different Velocities Using Real EMG

**Figure 5:**
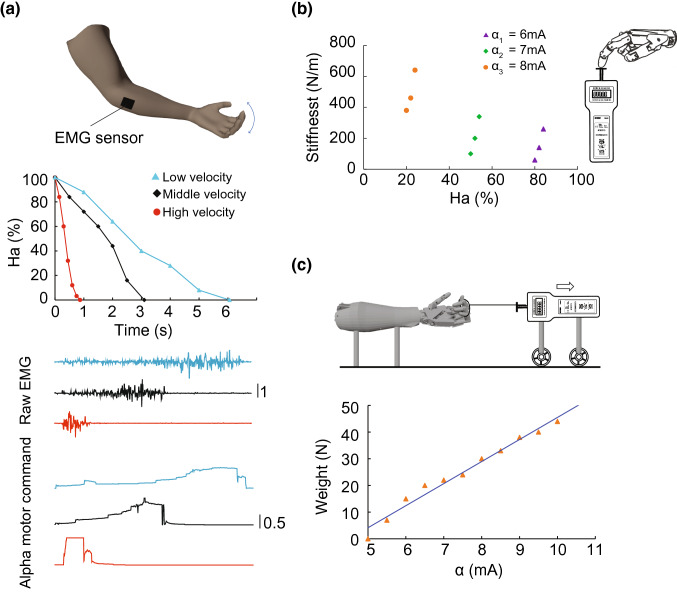
Experiment 2: operation of prosthetic hand. (a) A healthy subject activated the wrist EMG to close the hand from the resting-open to full-closure. Three different velocities were voluntarily chosen. (b) Varying alpha motor command produced different stiffness at the fingertip, 3 levels of alpha command were issued. The stiffness was also affected by the posture of the hand, 3 postures were sampled for each level of alpha command. (c) Test the weight-bearing ability of the prosthetic hand by sending various alpha motor command ($\alpha$). The hand could maintain up to 44 N of force, and the linear correlation between weight and $\alpha$ was significant.

In Experiment 2, we continued to verify whether a subject could close the prosthetic hand at different velocities, i.e. varying the speed when driving the hand from the resting posture to full closure. A healthy subject (male, age 30) controlled the prosthetic hand by contraction of the flexor carpi ulnaris muscle, wrist flexion was translated into hand closing on the prosthetic hand. In all three parts of experiment 2, the healthy subject was flexing his wrist with his fingers relaxed (Fig. 5a, top panel).

EMG was filtered using non-linear Bayesian filter and scaled to 0–20 mA. The subject was instructed to fully close the hand with either low, medium or high speed. As can be seen from Fig. 5a, the subject was capable of closing the hand swiftly in 0.87 s (purple), moderately in 3.11 s (orange), and more slowly in 6.06 s (blue). Raw EMGs recorded from the FCU of the subject are also shown. Alpha motor commands filtered from raw EMG using nonlinear Bayesian filter are also shown. Note the abrupt changes in the alpha motor commands, which are featured characteristics enabled by the Bayesian estimation.^7^

#### Quasi-static Stiffness at Different Gestures

We verified whether the hand could establish various stiffness at a chosen gesture. This was measured by having the finger producing a pressing force at the fingertip, and then recording the change in force after a small displacement. Wrist flexion was translated to the flexion of index finger (Fig. 5b). In specific, the model-based controller received an alpha motor command such that the finger was pressing against a force gauge (Model ELK-20, Elecall Co., Ltd., Wenzhou, China). Subsequently, the force gauge was elevated by 5mm and the change of force ($dF$) was recorded. The ratio of $dF/dH_{a}$ was calculated as the stiffness. We sampled three values of alpha motor command (6, 7, 8 mA) at 9 different apertures. As can be seen from Fig. 5b, the finger established a range of stiffness between 60 and 640 N/m, compared to 23 and 527 N/m as reported in human index finger.^10^ A comparison on the relative flexibility of stiffness, defined as $\left( {K_{\text{high}} - K_{\text{low}} } \right)/K_{\text{low}}$, shows that the prosthetic hand achieved 46.6% of the flexibility in stiffness of a human index finger.

#### Weight Bearing Ability

The weight bearing ability of the prosthetic hand was tested. Wrist flexion was translated to whole hand closing against a rope loop around the 4 fingers except for the thumb (Fig. 5c). A video demonstrating how the hand picked up a 2kg weight is available in the Supplementary Materials. The fingers were pulled together by a ring rope. We measured the maximal force when the ring rope was pulled off the hand. As can be seen from Fig. 5c, the hand could maintain up to 44 N of force, compared to 95.6 N maximum force of precision-grip force and 400 N maximum force of power-grip reported in human.^87^ The linear correlation between weight and alpha motor command was significant ($p < 0.01$, $R^{2} = 0.976$).

### Experiment 3: Human-Operated Performance with Object Interaction

#### Contacting a Deformable Object

**Figure 6:**
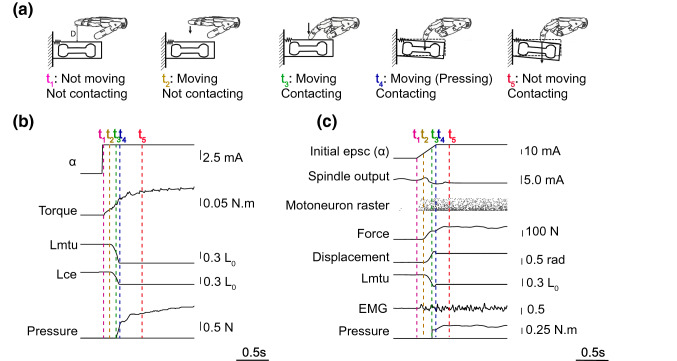
Experiment 3, the procedure of finger movement with object deformation. (a) Illustration of five key events when the index finger moved till it pressed against a bendable beam. (b) Signals from the model-based controller that helped identifying the progression of events. (c) Non-realtime software simulation using the same reflex model. Additional multi-scale information was available, including spindle output, motoneuron raster, simulated EMG, etc. The lineup of 5 events is similar to (b).

In Experiment 3, the index finger of the prosthetic hand was activated to press down a bendable bean. The finger was initially hovering at 1 cm above a bendable beam. Thereafter, a step alpha motor command was issued to the biomimetic model, leading to a series of events: change in motor torque (t1), change in muscle length (t2), contact of object (t3), end of deformation (t4), settlement of force (t5). An illustration of the five events can be seen in Fig. 6a. We issued a step signal of alpha (*a* = 6.5 mA) to the biomimetic model, and we measured the time of all five events given hovering distance of either 1 cm (Fig. 6b).

Due to limited bandwidth for data monitoring in hardware-in-the-loop applications, not all internal statuses were recorded. We simulated the contact of deformable beam in Simulink using the same model of reflex. Multi-scale information was available about spindle output, motoneuron spike raster, EMG, etc. A similar lineup of events (t1–t5) were identified from simulated data (Fig. 6c). The timing of events is shown in Table 2.

**Table 2:** Important events during finger flexion and the contact with object.

Event	Description	Timing hardware (s)	Timing simulation (s)
t1	Change in motor torque	0.00	0.00
t2	Change in muscle length	0.09	0.09
t3	Contact of object	0.19	0.24
t4	End of deformation	0.24	0.30
t5	Settlement of force	0.56	0.52

#### “Press-Without-Break” Performance in Amputee

**Figure 7:**
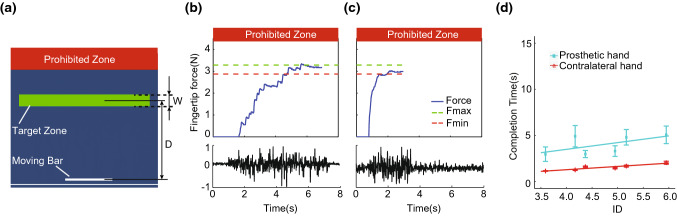
(a) The visual display during a typical “press-without-break” trial. The height of the white moving bar was linked to the finger pressure. The goal of the task was to escalate the moving bar and stay in the green target zone for 1 s. Task difficulty increases with longer distance (*D*) and narrower width (*W*). Red prohibited zone indicates object breakage once entered, which should be avoided at all times. (b) A representative trial of success of an amputee. The subject took 6.9 s to finish the task. EMG from FCU is shown. (c) A representative trial of success of the same amputee, using the index finger of the contralateral hand. The same task was accomplished with 3.0 s. (d) The linear relationship between completion time and index of difficulty (ID). The prosthetic hand achieved 49.4% information throughput as using the contralateral hand.

We tested how an amputee performed with the prosthetic hand in a “press-without-break” task. Using the above setup, the monitor displayed a moving bar, whose height (*D*) represented the finger pressure (Fig. 7a). In each trial, the subject was requested to raise the moving bar to a green target zone (*W*) as quickly as possible. A trial was considered successful once the moving bar had stayed in the target zone for 1 s. Above the target zone there was a prohibited zone in red. The amputee was informed that entering the prohibited zone meant breaking the virtual object, which should be avoided at all times. We chose a breakage threshold (lower bound of prohibited zone) of 4.4 N. Visual feedback was constantly available to subjects.

Each trial was associated with an Index of Difficulty (ID), formulated as ${\text{ID}} = { \log }_{2} \left( {2D/w} \right)$. A total of 6 different IDs were introduced in the experiment. Table 3 shows the target distances (*D*) and target width (*W*) corresponding to each ID.

**Table 3:** Distances (*D*) and widths (*W*) and the corresponding indices of difficulty (ID).

*D*(N)	*W*(N)	ID
1.8	0.1	5.17
1.8	0.2	4.17
1.8	0.3	3.59
3.1	0.1	5.95
3.1	0.2	4.95
3.1	0.3	4.37

We calculated the information throughput (TP) as a metric to quantify the performance,^5^ which reflects the speed-accuracy relationship commonly seen in human-machine interactions^23^:

$$ {\text{TP}} = \frac{1}{N}\mathop \sum \limits_{i = 1}^{N} {\text{ID}}_{i} /{\text{CT}}_{i} $$

where *N* = 6 is the number of IDs, and CT is the time for task completion in each trial.

The force and normalized EMG of a successful trial from an amputee (male, age 50, fore-arm amputation, consented as approved by the Ethics Committee of Human and Animal Experiments of the Med-X Research Institute of Shanghai Jiao Tong University) are shown in Fig. 7b; data of a successful trial using his contralateral hand (index finger, no prosthetic hand) are shown in Fig. 7c. The information throughput was 1.561 bit/s using prosthetic hand, compared to 3.160 bit/s using the contralateral hand (Fig. 7d).

## Discussion

We have designed a model-based controller that enables biomimetic reflex on cable-driven prosthetic hands. The biomimetic reflex comprises of spiking neurons, skeletal muscles, muscle spindles, and monosynaptic spinal circuitry. The model-based controller interfaces between surface EMG and an electric motor that pulls the cables. We have also shown how to reconstruct proprioceptive information based on readouts from the motor. The closed-loop behavior of the reflex-enabled prosthetic hand has been tested in six experiments. Results suggest that the hand could achieve expected behaviors, including moving with different destinations or velocities, pressing against a bendable beam, grasping paper or steel cups, etc. This study verified the feasibility of enabling biomimetic reflex in the real-time control of cable-driven prosthetic hand.

Previous work has demonstrated the capability of simulated monosynaptic reflex for replication of human-like movements in health and disease,^51^,^61^ which comprises of spiking neurons, muscle spindles, synapses, skeletal muscles, and monosynaptic spinal circuitry. The “virtual reflex” provides the foundation for model-based control in this study, but it is repurposed for translation of motor command into expected muscle force and hand stiffness. We adhere to a criterion that all parameters with biomimetic reflex should be traceable to mammalian neurophysiology. In future applications, however, parameters can deviate from normal physiology if proven more advantageous in motor performance. One advantage of neuromorphic hardware is that it allows for flexible trial-and-error against various model configurations. Models in the form of differential equations (e.g. spindle) are computationally expensive and generally difficult to run in real-time,^2^,^62^ making it difficult to be integrated in the closed-loop control of prosthetic hand. Acceleration of model evaluation is another advantage from neuromorphic hardware.

The model-based controller aims to provide an informational context compatible with the amputee. The model attempts that the alpha motor commands (inferred from EMG) operate through a machinery (motoneurons, muscles, spindles, etc.) as if there were no amputation. Both the healthy subject and amputee were given 10 minutes to familiarize with the prosthetic control, during which time the subjects may look at the prosthetic hand and the object, and adjust their commands if they saw any failure. In order to clarify the contribution of model-based control separate from the visual feedback, it might be necessary to perturb the vision in future experiments.

This study has by no means exhausted the voluminous possibilities of non-biomimetic approaches for prosthetic control (15,21,33,35,36,69,73,77,88 for an incomplete list). In fact, most of existing algorithms focused on the feedforward components, or introducing more loops to allow for complex dynamics in the closed-loop behavior. This study adopted the most parsimonious architecture for closed-loop control,^47^ and reduced the question to whether computationally intensive—but more biomimetic—models could enhance the performance. One explanation for the observed improvements in performance with biomimetic models is that they maintain a neurocompatibility between the device and amputee.^49^ Alpha motor commands from the peripheral nervous system are supposed to activate a machinery with muscle properties, spindle feedback, etc. A controller with neuromorphic models, therefore, provided such a virtual environment that is compatible with the original intention of subjects before amputation. It is also noteworthy that all neuromorphic models implemented in this study are, in essence, mathematical formulations of high-order nonlinear systems.^48^,^56^,^61^ Biomimetic models do not contradict the linear ones in principle of control, but rather they showed where the complexity should reside. In particular, impedance control measures position and generates force, which shares the same input-output structure as our model-based controller, and therefore impedance control strategies are reasonable contenders when benchmarking the model-based controller.

Only one afferented muscle was enabled on the index finger for its flexion, leaving the extension passively stretched by a spring. This setup suffices in engineering to produce whole-hand grasping when all five fingers are all configured similarly. But it lacks the biological realism that both flexion and extension should be separately actuated by a pair of antagonistic muscles. In practice, the flexion-only setup requires a constant EMG activity whenever the amputee wants to exert a force, meaning it also loses the capability of resetting the resting posture. Thus, it might be crucial to enable two autonomous muscles for each joint, such that the controller both achieves higher level of biomimicry and solves the practical problem of posture resetting. The model-driven strategy of control is not limited to cable-driven prosthetic hands. For non-cable-driven hands, the proprioceptive information can be reconstructed in a similar way from the angle transducers. It is noteworthy, however, that most non-cable-driven designs use non-backdrivable transmissions (e.g. gears or link-mechanisms), and therefore the controller would not be directly applicable without addressing the back-drivability beforehand.

## Electronic supplementary material

Below is the link to the electronic supplementary material.

Supplementary material 1 (DOCX 71 kb)

Supplementary material 2 (MP4 1923 kb)

Supplementary material 3 (MP4 1391 kb)

## Abbreviations

$L_{\text{mtu}}$: Length of musculotendinous unit
$L_{\text{ce}}$: Length of contractile element
$H_{\text{a}}$: Opening aperture of prosthetic hand
$\theta_{\text{motor}}$: Rotational angle of DC motor
$K_{\text{se}}$: The series elastic component
$K_{\text{pe}}$: The parallel elastic component
$K_{\alpha }$: The active component in modified Hill muscle model
$F_{L}$: Length-sensitive term, muscle model
$F_{V}$: Velocity-sensitive term, muscle model
$F_{A}$: Active muscle force
$V_{\text{m}}$: Maximum contraction velocity of muscle
$L_{t}$: Length of passive element
$F_{0}$: Peak active force
$F_{1}$: Active force at maximum contraction velocity of muscle
FCU: Flexor carpi ulnaris
EPSC: Excitatory post-synaptic current
VLSI: Very large scale integrated circuit
ID: Index of difficulty
CT: Completion time
TP: Throughput
