# Supplementary material for: Neuromorphic Model of Reflex for Realtime Human-Like Compliant Control of Prosthetic Hand
Source: Ann Biomed Eng. 2020 Aug 20;49(2):673–88. doi: 10.1007/s10439-020-02596-9 (PMC7851042; doi:10.1007/s10439-020-02596-9)
Supplement: Supplementary file 1 — Supplementary material 1 (DOCX 71 kb) [file 10439_2020_2596_MOESM1_ESM.docx]

# Appendix

## Mathematical description of the muscle model ^74^:

### Input variables

$u_{m1}=L_{ce}$, muscle length；

$u_{m2}=\dot{L}_{ce}$, muscle velocity；

$u_{m3}=A$, muscle activation；

### Output variables

$y_{m}=T$, muscle force；

### State variables

$x_{m}=T,$ muscle force.

### Parameters

**Table S1 Muscle model parameters**

| Parameter | Parameter Definition | Value |
| --- | --- | --- |
| *K_SE_* | Stiffness of the series elastic (SE) element | 136 g/cm |
| *K_PE_* | Stiffness of the parallel elastic (PE) element | 75 g/cm |
| *b* | viscosity of the fluid in its cavity | 50g.s/m |

### Formula

| $\dot{x}_{m}$ | $=\frac{K_{SE}}{b}\left( K_{PE}\left( u_{m1}-{u_{m1}}^{*} \right)+bu_{m2}-\left( 1+\frac{K_{PE}}{K_{SE}} \right)x_{m}+u_{m3} \right)$ |
| --- | --- |
| $x_{m}\left( 0 \right)$ | $=0$ |
| ${u_{m1}}^{*}$ | $=u_{m1}(0)=0.8$ |
| $y_{m}$ | $=x_{m}$ |

## Mathematical description of the neuron model ^41^:

### Input variables

$u_{n1}=I$, postsynaptic currents；

### Output variables

$y_{n}=v$, membrane potential；

### State variables

$x_{n1}=v，$membrane potential;

$x_{n2}=u$, membrane recovery variable.

### Parameters

**Table S2 Neuron model parameters**

| Parameter | Parameter Definition | Value |
| --- | --- | --- |
| *a* | the time scale of the membrane recovery variable u | 0.02 |
| *b* | the sensitivity of the recovery variable u to the subthreshold fluctuations of the membrane potential v | 0.2 |
| *c* | after-spike reset value of the membrane potential caused by the fast high-threshold K+ conductances | -65mV |
| *d* | after-spike reset of the recovery variable caused by slow high-threshold Na+ and K+ conductances | 2 |

### Formula

| $\dot{x_{n1}}$ | $=0.04{x_{n1}}^{2}+5x_{n1}+140-x_{n2}+u_{n1}$ |
| --- | --- |
| $\dot{x_{n2}}$ | $=a(bx_{n1}-x_{n2})$ |
| $x_{n1}\left( 0 \right)$ | $=-70mV$ |
| $x_{n2}\left( 0 \right)$ | $=-14$ |
| $y_{n}$ | ${=x}_{n1}$ |

If $x_{n1}\geq30mV,$ then $\left\{ \begin{aligned} x_{n1}\leftarrow c \\ x_{n2}\leftarrow x_{n2}+d \end{aligned} \right.$

## Mathematical description of the spindle model (bag 1) ^54^:

### Input variables

$u_{s1}=\Gamma$, gamma signals；

$u_{s2}=L_{ce}$，Fascicle length；

$u_{s3}=\dot{L}_{ce}$，Fascicle velocity；

### Output variables

$y_{s}=f_{afferent}$, afferent signals of spindle model；

### States variables

$x_{s1}$, spindle activation—$f_{act}$；

$x_{s2}$, intrafusal muscle length—$L_{PR}$；

$x_{s3}$, intrafusal muscle velocity—$\dot{L}_{PR}$.

### Parameters

**Table S3 Spindle model parameters**

| Parameter | Parameter Definition | Value (bag_1_) | Value (bag_2_) | Value (chain) |
| --- | --- | --- | --- | --- |
| *K_SR_* | Sensory region spring constant [FU/L_0_] | 10.4649 | 10.4649 | 10.4649 |
| *K_PR_* | Polar region spring constant [FU/L_0_] | 0.1500 | 0.1500 | 0.1500 |
| *M* | Intrafusal fiber mass [FU/(L_0_/s^2^)] | 0.0002 | 0.0002 | 0.0002 |
| *B_0_* | Passive damping coefficient [FU/(L_0_/s)] | 0.0605 | 0.0822 | 0.0822 |
| *B_1_* | Coef. of damping due to dyn. fusimotor input [FU/(L_0_/s)] | 0.2592 |  |  |
| *B_2_* | Coef. of damping due to stat. fusimotor input [FU/(L_0_/s)] |  | -0.0460 | -0.0690 |
| $\Gamma_{1}$ | Coef. of force generation due to dyn. fusimotor input [FU] | 0.0289 |  |  |
| $\Gamma_{2}$ | Coef. of force generation due to stat. fusimotor input [FU] |  | 0.0636 | 0.0954 |
| *G* | Term relating the sensory region’s stretch to afferent firing | 20000 | 10000 | 10000 |
| *R* | Fascicle length below which force production is zero (L_0_) | 0.46 | 0.46 | 0.46 |
| *L_PR0_* | Sensory region rest length (L_0_) | 0.04 | 0.04 | 0.04 |
| *L_PR0_* | Polar region rest length (L_0_) | 0.76 | 0.76 | 0.76 |
| *Ω (freq)* | Constant relating the fusimotor frequency to activation | 60 | 60 | 90 |
| *τ* | Low-pass filter time constant | 0.149 | 0.205 |  |

### Formula

| $\dot{x}_{s1}$ | ${=f}_{d1}\left( t,x_{s1},u_{1} \right)$ |
| --- | --- |
| $\dot{x}_{s2}$ | ${=f}_{d2}\left( t,x_{s2},u_{2} \right)$ |
| $\dot{x}_{s3}$ | ${=f}_{d3}\left( t,x_{s3},u_{s1},u_{s2},u_{s3} \right)$ |
| $x_{s1}\left( 0 \right)$ | $=0$ |
| $x_{s2}\left( 0 \right)$ | $=0.8$ |
| $x_{s3}(0)$ | $=0$ |
| $y_{s}$ | ${=f}_{o}\left( t, x_{s1},x_{s2},x_{s3},u_{s1},u_{s2},u_{s3} \right)$ |

### Synapse model ^28^

When a pre-synaptic neuron spikes, i.e. S(0)=1, an excitatory synapse subsequently issues an Excitatory Post-Synaptic Current (EPSC) that drives the post-synaptic neuron. Neural recording of hair cells in rats provided evidence that the time profile of EPSC can be well characterized using the equations below:

$$I\left( t \right)=\left\{ \begin{aligned} V_{m}\times\left( e^{-\frac{t}{\tau_{d}\tau_{m}}}-e^{-\frac{t}{\tau_{r}\tau_{m}}} \right) if t\geq0 \\ 0 otherwise \end{aligned} \right.$$

The key parameters in a synapse model is the time constants for rising (τr) and decaying (τd). In our emulation τr = 0.001 s and τd = 0.003 s.
